# Metabolic Hormones in Schizophrenia Patients with Antipsychotic-Induced Metabolic Syndrome

**DOI:** 10.3390/jpm12101655

**Published:** 2022-10-05

**Authors:** Anastasiia S. Boiko, Irina A. Mednova, Elena G. Kornetova, Anastasiia A. Goncharova, Arkadiy V. Semke, Nikolay A. Bokhan, Svetlana A. Ivanova

**Affiliations:** 1 Mental Health Research Institute, Tomsk National Research Medical Center of the Russian Academy of Sciences, Aleutskaya Str., 4, 634014 Tomsk, Russia; 2 Department of Psychiatry, Addictology and Psychotherapy, Siberian State Medical University, Moskovsky Tract, 2, 634050 Tomsk, Russia

**Keywords:** schizophrenia, metabolic syndrome, antipsychotics, body mass index, insulin, leptin, ghrelin

## Abstract

Metabolic syndrome (MetS) is a common complication of schizophrenia that is quite exacerbated by long-term use of (atypical) antipsychotics. The mechanism of MetS has neuronal, neuroendocrine, and neuroimmunological components and shows some overlap with those of aspects of schizophrenia. We examined 195 patients with schizophrenia (90 with and 105 without MetS) for the association of serum levels of ghrelin, insulin, and leptin with metabolic abnormalities. Serum glucose levels and lipid profiles were routinely measured with colorimetric enzymatic methods and hormone levels with multiplex analyzers. Leptin levels were highly significantly increased (*p* < 0.001) in people with MetS (9.966 [5.882; 21.496] vs. 6.35 [2.005; 11.753], Me [Q1; Q3]) and ghrelin levels were actually significantly decreased (*p* = 0.045). Insulin levels did not differ significantly between those with and without MetS (*p* = 0.162). In Spearman’s correlation analysis between the hormone levels, body characteristics, and biochemical parameters, significant correlations were seen somewhat more often in people without MetS than in those with MetS and also less often for ghrelin than for the other hormones. We conclude that evidence exists for a role in the development of MetS especially for leptin, but that less is supporting a role for ghrelin.

## 1. Introduction

Schizophrenia, whose global age-adjusted prevalence in 2016 was estimated at 0.28% [1], may not be a very common disease, but it is so disabling that it accounts for 12.2% [9.6–15.2%] of all disability-adjusted life-years (DALYs) of the total of mental illness worldwide in 2019 [2]. As with many other mental illnesses, a relatively high percentage of patients with schizophrenia also have metabolic disorders, particularly metabolic syndrome [3], which is a combination of central obesity, dyslipidemia, hyperglycemia, and hypertension [4]. The prevalence of MetS in patients with schizophrenia varies substantially in different studies depending on their country (see [5]), but also within our own region are there significant differences between the hospitals involved [6].

Antipsychotics are undoubtedly the most commonly used to treat the symptoms of an exacerbation of schizophrenia and to prevent a new outbreak of the disease. Although the prevalence of (components of) MetS is also increased in antipsychotic-naive patients with schizophrenia [7,8], the prevalence is higher in people with schizophrenia treated with antipsychotics [9]. Regarding the former, there is evidence that schizophrenia and MetS share some of the same risk factors and that these are interrelated [10,11]. Relatively recently, research has begun to explore how metabolic disorders may affect psychopathological or cognitive outcomes in severe mental disorders [12]. Regarding the latter, antipsychotics differ in their pharmacological profile and in their cardiometabolic properties [13]. Taking these differences into account, the use of antipsychotics is at least among the strongest contributors to weight gain and the development of metabolic disorders, including diabetes, in patients with mental disorders [14,15].

In our opinion, there is little doubt that the connection between the (psycho)pathological and (psycho)pharmacological effects has its material basis in neuronal, neuroendocrine, and neuroimmunological processes. We have therefore explored the various components in more detail in the past. Regarding the connection with neurotransmitter function, we have limited ourselves to genetic variants of the 5-hydroxytryptamine and dopamine receptors [16,17]. The significance of entities with an immunomodulatory function was also studied in detail: apolipoproteins [18] and a variety of cytokines [19,20]. Most extensively, we examined the possible involvement of endocrine regulators: thyroid and steroid hormones [5,21] and other hormonal regulators of metabolism such as adipokines and insulin [19,22,23]. The results are difficult to summarize in a few lines, but they support the previously formulated idea that both changes in neurotransmission (serotonergic and dopaminergic), neuroinflammatory processes, and endocrine regulation are involved in the onset of metabolic syndrome.

In one of our previous studies [19], we found significantly increased leptin levels in schizophrenia patients with compared to those without MetS. Leptin levels correlated with several metabolic parameters, both in patients with and without MetS, including body-fat percentage, total fat fold, and body mass index (BMI). Multiple regression analysis showed multiple associations for leptin. For the other adipocytokines measured, this association was absent or much less present. Increased leptin levels in the presence of MetS were also found by others in this population [10]. It has been suggested that the increase in leptin levels is primarily a result of the increase in body weight and not a direct effect on leptin metabolism [24]. However, even partially independent of BMI and therapy, leptin is elevated in patients with schizophrenia compared to healthy subjects [25]. That the leptin elevation in antipsychotic use is a consequence rather than a cause of the increase in adipocytes may be a logical thought, but in a recent study we also found that the functional polymorphism rs3828942 of the gene encoding leptin (*LEP*) is strongly associated with the presence of MetS [23]. Leptin is indeed produced by mature adipocytes of the white adipose tissue, but the association with the polymorphism suggests that its production is also actively regulated, given its role in regulatory processes other than those of nutrition [23]. Ghrelin is another hormonal player in the involved field of weight and hunger regulation [23,24,26], although the existence of an acylated form makes the situation somewhat more complex [27]. Ghrelin is produced primarily in the stomach and is best known for promoting appetite [23]. Interestingly, ghrelin is (also) involved in circadian regulation for food intake motivation [23].

In our earlier study [19], we did not yet include ghrelin. In the current research, we measure the levels of leptin, ghrelin, and insulin in a larger patient population and correlate the findings with (other) biochemical parameters, waist circumference, and body mass index (BMI).

## 2. Materials and Methods

### 2.1. Patients

The study was conducted in accordance with the Declaration of Helsinki (1964, revised in Fortaleza, Brazil, October 2013) and according to a protocol reviewed and approved by the Bioethics Committee of the Mental Health Research Institute of the Tomsk National Research Medical Center of the Russian Academy of Sciences (Tomsk NRMC) (Protocol 187, approval on 24 April 2018). After obtaining informed consent, we recruited 195 patients with schizophrenia who were treated in the clinics of the Mental Health Research Institute of the Tomsk NRMC, the clinics of the Siberian State Medical University, the Tomsk Clinical Psychiatric Hospital, the Kemerovo Regional Clinical Psychiatric Hospital, and the N.N. Solodnikova Clinical Psychiatric Hospital of Omsk in the Russian Federation.

The main criteria for inclusion of patients in the study were a verified diagnosis of schizophrenia according to the ICD-10 (International Statistical Classification of Diseases and Related Health Problems, 10th Revision), an age of 18–65 years, the granting of informed consent, and absence of severe organic pathology or somatic disorders at the stage of decompensation. 

The antipsychotics and concomitant therapy the patients were receiving at the time of the study (drugs, doses used, and duration of current drug use), as well as the antipsychotics and concomitant somatic therapy used during the last 6 months, were assessed. A chlorpromazine equivalent (CPZeq) was used in the study to standardize the dose, efficacy, and side effects of antipsychotics [28]. The severity of psychopathology was measured by applying the positive and negative syndrome scale (PANSS) [29].

MetS was diagnosed according to the International Diabetes Federation (IDF) criteria [4].

### 2.2. Biochemical Analysis

In the studied patients, blood samples were collected by antecubital venipuncture into BD Vacutainer tubes containing an activator of coagulation (SiO_2_). To obtain serum for measurement of biochemical parameters, blood was centrifuged at 2000 rcf at 4 °C for 20 min. Serum samples were stored at −80 °C until analyzed.

Biochemical measures for the lipid spectrum, including glucose, total cholesterol, triglycerides, and lipoproteins, were measured by colorimetric enzymatic method using commercial kits from “Cormay” (Łomianki, Poland). 

Concentrations of leptin, ghrelin, and insulin were determined on the multiplex analyzers Magpix and Luminex 200 (Luminex, Austin, TX, USA) using xMAP^®^ technology, based in the Core Facility “Medical genomics” (Tomsk NRMC). The HMHEMAG-34K panel of MILLIPLEX^®^ MAP (Merck, Darmstadt, Germany) was used to determine the levels of hormones. The detected information is processed by special Luminex xPONENT^®^ software, with subsequent export of data to the MILLIPLEX^®^ Analyst 5.1 program.

### 2.3. Statistical Analysis

Statistical analysis was performed with SPSS software, version 23.0 (IBM, Armonk, NY, USA). Student’s *t*-test, Pearson’s chi-square test, Mann–Whitney’s U-test, and Fisher’s exact criterion were used. The choice of one or the other methods of analysis was made on the basis of the extent to which the criteria for their applicability (quantitative or qualitative characteristic, agreement with the law of normal distribution, and equality of variances) were met. The results are presented in the form of mean ± SD (SD—the standard deviation) for data that conform to the law of normal distribution, and in the form of Me [Q1; Q3] (median, lowest/first and highest/third quartile) for data that do not conform to this distribution. Spearman’s correlation analysis was used to evaluate the relationships between the studied parameters. The critical significance level was 0.05.

## 3. Results

### 3.1. Patient Characteristics

A total of 195 patients receiving antipsychotic therapy were examined. Metabolic syndrome was diagnosed in 90 patients (46.2%). Table 1 presents the main demographic and clinical parameters of the studied patient groups.

The gender distribution of the patients was equivalent in the study groups without and with MetS. The patients with MetS were significantly older (*p* < 0.001), and the duration of the condition was longer in these patients than in the comparison group (*p* < 0.001). The study groups also showed significant differences in body mass index and waist circumference (*p* < 0.001).

### 3.2. Biochemical Measures

The results of the biochemical analysis of the glucose levels and lipid spectrum are shown in Table 2.

A significant increase in the concentrations of glucose, triglycerides, VLDL (*p* < 0.001), and total cholesterol (*p* = 0.038) was observed in patients with MetS. A decrease in HDL level was also observed in these patients compared to the group without MetS (*p* < 0.001).

### 3.3. Hormone Levels

As a result of our study, a statistically significant increase in leptin concentration (*p* < 0.001) and a decrease in ghrelin levels (*p* = 0.045) were observed in patients with schizophrenia with MetS compared to the group without MetS (Table 3).

### 3.4. Correlation Analysis

Spearman’s correlation analysis was carried out to assess the interrelationships of the studied parameters among themselves in groups of patients without MetS (Table 4) and with MetS (Table 5).

Ghrelin has one weak negative correlation with waist circumference in patients without MetS. Insulin has weak positive correlations with BMI, waist circumference, triglycerides, VLDL, and leptin. Leptin correlates with BMI at an average level in schizophrenia patients without MetS and also has weak correlations with waist circumference, triglycerides, and VLDL.

Fewer significant correlations between the studied parameters were found in the group of patients with MetS. Insulin and leptin are weakly correlated with waist size and glucose levels, and with each other. Leptin also has a weak correlation with BMI. There are no significant correlations between ghrelin and other parameters in the group with MetS.

## 4. Discussion

In the current study of people with schizophrenia, we found highly significantly higher leptin levels in the serum of 90 patients with MetS than in those 105 without MetS. Ghrelin levels were less conclusively different and insulin levels were not. In a correlation analysis of hormone levels, body characteristics, and relevant biochemical parameters, we observed much less of an association for ghrelin levels than for the other two hormones.

Patients with MetS were significantly older and sick longer than those without MetS. Although drug-naive people with schizophrenia are more likely to have MetS than healthy individuals [7,8], and therefore the schizophrenic disease process itself may influence the prevalence of MetS, no conclusions should be drawn from this in our case. Age itself is an important factor [30,31], and probably disease duration also correlates with the duration of exposure to (atypical) antipsychotics. Sherk et al. [32] found 35% higher serum leptin levels in older postmenopausal women compared with younger premenopausal, but this difference evaporated after controlling for fat mass.

Our results regarding serum levels of leptin correspond well with our findings in a previous study of a more limited size [19]. There, too, we found significantly elevated leptin levels in people with MetS and, in addition, significantly correlated leptin levels (similarly dependent on the presence of MetS) with BMI, waist circumference, glucose, triglyceride, and insulin levels. Increased serum leptin concentrations have been found more frequently in patients with schizophrenia, especially when taking antipsychotics [24,25,33,34], which is consistent with our results. Leptin, under physiological conditions, increases energy consumption (including by enhancing insulin action and increasing sympathetic activity) and inhibits appetite [35,36]. The cerebral effects are achieved by activating part of the pro-opiomelanocortin (POMC) neurons of the arcuate nucleus (ARC) of the hypothalamus (appetite) and the nucleus of the tractus solitarius (NTS) in the lower brainstem (sympathetic activity) [36]. This cerebral leptin–melanocortin pathway is an important neuroendocrine regulator of energy homeostasis [3]. It is notable that, with overfeeding, a situation of hyperleptinaemia quickly develops, accompanied by leptin, ghrelin, and insulin resistance and also by the blocking of the intracerebral effects [23,35,37]. The cause of this resistance and its significance in the development of obesity is not yet fully understood [23,37]. Leptin is produced by adipocytes, and serum levels correlate well with the amount of adipose tissue in the body [19,38]. Therefore, the idea may arise that the increase in leptin levels in MetS is of little significance because it is only another measure of the increased amount of fat in the body [24]. With leptin resistance, the feedback control mechanism no longer works. Nevertheless, there is probably more to it [25]. For other adipocytokines that are also produced by fat cells, a link was not observed in our previous study [19]. According to the literature, the leptin–melanocortin pathway plays a role in regulation by influencing neurogenesis and neuroplasticity in the hippocampus and cortical structures, as well as in regulation of the hypothalamic–pituitary–adrenal (HPA) axis and immune system activity [39]. It is not certain, even somewhat unlikely, that resistance to the cerebral effects of leptin is equally strong everywhere. The polymorphism rs3828942 of *LEP* mentioned in the introduction is not so important for homeostatic energy regulation, as it is significant for sleep and anxiety regulation [23]. We would argue that the influence of hyperleptinaemia on the activity of reward-seeking and distress-avoiding processes controlled by the dorsal diencephalic connection system (habenula) [40,41,42] should also be critically considered.

Despite the fact that we found almost no significant correlations between ghrelin and the other measured parameters, there is a significant decrease in ghrelin concentration in schizophrenia patients with MetS. In a meta-analysis, reduction in ghrelin levels was found in association with the initiation of olanzapine, a drug known to cause body weight gain [26]. In studies with other antipsychotics, the results are difficult to interpret [43]. In the general population, an association between lowered ghrelin and metabolic syndrome has been found previously [44,45]. Thus, according to these studies and similar to our data, lowered ghrelin levels might well be an important predictor of the development of the MetS in patients with schizophrenia. How that is supposed to work then is very unclear. In any case, the impact of ghrelin seems much less than that of leptin. Preclinical research has also shown that increased serum leptin levels, but not those of ghrelin, may precede weight gain induced by olanzapine [46]. In addition, metabolic disorders and disturbances in metabolic hormone regulation may be more primarily associated with schizophrenia itself [47].

One of the possible causes of disturbances in the concentrations of metabolic hormones may be the polymorphisms of the genes encoding them [48]. Previously, we have identified an association of genotypes and alleles of the rs3828942 of *LEP* with the development of metabolic syndrome in patients with schizophrenia receiving antipsychotic therapy [23]. The genotype AA and the allele A of the rs3828942 have a predisposing effect on the development of MetS (OR1 = 2.06, 95% CIs: 1.16–3.64; OR2 = 1.4, 95% CIs: 1.05–1.87).

The limitation of our study is the lack of information on antipsychotic treatment throughout the illness. The use of antipsychotics with different mechanisms of action and the duration of their use may play a role in the development of MetS.

## 5. Conclusions

In our study, we found abnormalities in the concentrations of leptin and ghrelin in schizophrenia patients with metabolic syndrome. Leptin levels correlated much more robustly with relevant body characteristics and metabolic parameters than those of ghrelin. Along which pathway leptin and ghrelin contribute to the development of metabolic syndrome in people with schizophrenia is uncertain. It is recommended that future research should include not only their involvement in energy metabolism but also in higher forms of behavioral regulation.

## Figures and Tables

**Table 1:** Demographic and clinical parameters of the studied patient groups.

Parameter		Patients without MetS *n* = 105 (53.8%)	Patients with MetS *n* = 90 (46.2%)	*p*-Value
Gender	Female, *n* (%)	53 (50.48%)	54 (60%)	0.145
	Male, *n* (%)	52 (49.52%)	36 (40%)	
Age, years (Me [Q1; Q3])		32 [26.75; 36.25]	44.5 [33.75; 52.25]	<0.001 *
Duration of disorder, years (Me [Q1; Q3])		8 [3; 14]	16 [9; 22]	<0.001 *
CPZeq, dose (Me [Q1; Q3])		322.5 [199.95; 520]	450 [208.74; 754.5]	0.085
PANSS, total score (Me [Q1; Q3])		97 [86; 106]	101 [87; 110]	0.16
BMI, (Me [Q1; Q3])		24 [21.9; 28.4]	31.15 [26.9; 35.575]	<0.001 *
Waist circumference, cm (Mean ± SD)		85.83 ± 13.216	104.82 ± 12.079	<0.001#

Notes: MetS—metabolic syndrome. CPZeq—chlorpromazine equivalents. BMI—body mass index. Me [Q1; Q3]—median and quartiles. Mean ± SD—mean and standard deviation. *—significance of differences according to the Mann–Whitney U-test. #—significance of differences according to Student’s *t*-test.

**Table 2:** Concentration of glucose and lipid spectrum in blood serum of patients without and with metabolic syndrome (mean ± SD; Me [Q1; Q3]).

Parameter	Patients without MetS *n* = 105	Patients with MetS *n* = 90	*p*-Value
Glucose, mmol/L	4.69 ± 0.545	5.21 ± 0.79	<0.001 #
Triglycerides, mmol/L	1.07 [0.8; 1.5]	1.99 [1.56; 2.5]	<0.001 *
Total cholesterol, mmol/L	4.22 [3.76; 4.94]	4.64 [4.015; 5.39]	0.038 *
HDL, mmol/L	1.1 [0.9; 1.3]	0.87 [0.7; 1.02]	<0.001 *
LDL, mmol/L	2.64 [2.23; 3.225]	2.83 [2.075; 3.505]	0.485
VLDL, mmol/L	0.5 [0.36; 0.68]	0.91 [0.77; 1.14]	<0.001*

Notes: MetS—metabolic syndrome. HDL—high-density lipoproteins. LDL—low-density lipoproteins. VLDL—very-low-density lipoproteins. *—significance of differences according to the Mann–Whitney U-test. #—significance of differences according to Student’s *t*-test.

**Table 3:** Insulin, ghrelin, and leptin concentrations in blood serum of schizophrenia patients without and with metabolic syndrome (Me [Q1; Q3]).

Hormones	Patients without MetS *n* = 105	Patients with MetS *n* = 90	*p*-Value
Insulin, pg/mL	557.74 [442.71; 952.67]	698.36 [465.26; 917.825]	0.162
Ghrelin, pg/mL	24.02 [17.41; 33.385]	22.83 [15.31; 26.12]	0.045 *
Leptin, ng/mL	6.35 [2.005; 11.753]	9.966 [5.882; 21.496]	<0.001 *

Notes: MetS—metabolic syndrome. *—significance of differences according to the Mann–Whitney U-test.

**Table 4:** Spearman’s correlation analysis between hormones, body mass index and biochemical parameters of the lipid spectrum in patients without metabolic syndrome.

Parameter		Ghrelin	Insulin	Leptin
BMI	Po	−0.219	0.287	0.610
	*p*-value	0.053	0.010 *	<0.001 *
Waist circumference	Po	−0.270	0.245	0.432
	*p*-value	0.015 *	0.027 *	<0.001 *
Glucose	Po	−0.206	−0.109	0.010
	*p*-value	0.050	0.303	0.927
Triglycerides	Po	−0.094	0.320	0.338
	*p*-value	0.373	0.002 *	<0.001 *
Total cholesterol	Po	−0.198	−0.018	0.193
	*p*-value	0.061	0.869	0.068
HDL	Po	−0.128	−0.280	0.072
	*p*-value	0.225	0.007 *	0.496
LDL	Po	−0.168	0.014	0.125
	*p*-value	0.113	0.896	0.240
VLDL	Po	−0.063	0.291	0.390
	*p*-value	0.557	0.005 *	<0.001 *
Ghrelin	Po	1	0.044	0.067
	*p*-value		0.653	0.496
Insulin	Po	0.044	1	0.266
	*p*-value	0.653		0.006 *
Leptin	Po	0.067	0.266	1
	*p*-value	0.496	0.006 *	

Notes: BMI—body mass index. HDL—high-density lipoproteins. LDL—low-density lipoproteins. VLDL—very-low-density lipoproteins. Po—Spearman’s coefficient. * *p*-value—significance of differences less 0.05.

**Table 5:** Spearman’s correlation analysis between hormones, body mass index, and biochemical parameters of the lipid spectrum in patients with metabolic syndrome.

Parameter		Ghrelin	Insulin	Leptin
BMI	Po	−0.156	0.167	0.447
	*p*-value	0.160	0.129	<0.001 *
Waist circumference	Po	−0.152	0.271	0.310
	*p*-value	0.159	0.011 *	0.003 *
Glucose	Po	0.042	0.266	0.257
	*p*-value	0.695	0.011 *	0.014 *
Triglycerides	Po	−0.132	0.143	0.156
	*p*-value	0.218	0.180	0.141
Total cholesterol	Po	0.029	0.131	0.113
	*p*-value	0.789	0.217	0.287
HDL	Po	−0.047	0.027	0.190
	*p*-value	0.660	0.802	0.073
LDL	Po	0.208	0.191	0.090
	*p*-value	0.096	0.125	0.473
VLDL	Po	−0.117	0.076	0.218
	*p*-value	0.357	0.549	0.081
Ghrelin	Po	1	−0.071	−0.034
	*p*-value		0.508	0.754
Insulin	Po	−0.071	1	0.415
	*p*-value	0.508		<0.001 *
Leptin	Po	−0.034	0.415	1
	*p*-value	0.754	<0.001 *	

Notes: BMI—body mass index. HDL—high-density lipoproteins. LDL—low-density lipoproteins. VLDL—very-low-density lipoproteins. Po—Spearman’s coefficient. * *p*-value—significance of differences less 0.05.

## Data Availability

The datasets are available on reasonable request to Prof Svetlana A. Ivanova (ivanovaniipz@gmail.com), following approval of the Board of Directors of the MHRI, in line with local guidelines and regulations.
